# Immunoglobulin Tau Heavy Chain (IgT) in Flounder, *Paralichthys olivaceus*: Molecular Cloning, Characterization, and Expression Analyses

**DOI:** 10.3390/ijms17091571

**Published:** 2016-09-17

**Authors:** Yang Du, Xiaoqian Tang, Wenbin Zhan, Jing Xing, Xiuzhen Sheng

**Affiliations:** 1 Laboratory of Pathology and Immunology of Aquatic Animals, KLM, Ocean University of China, 5 Yushan Road, Qingdao 266003, China; duyang0422@sina.com (Y.D.); tangxq@ouc.edu.cn (X.T.); wbzhan@ouc.edu.cn (W.Z.); xingjing@ouc.edu.cn (J.X.); 2 Function Laboratory for Marine Fisheries Science and Food Production Processes, Qingdao National Laboratory for Marine Science and Technology, No. 1 Wenhai Road, Aoshanwei Town, Jimo, Qingdao 266071, China

**Keywords:** tau immunoglobulin (IgT), flounder (*Paralichthys olivaceus*), gene cloning, expression dynamic, mucosal immunity

## Abstract

Immunoglobulin tau (IgT) is a new teleost immunoglobulin isotype, and its potential function in adaptive immunity is not very clear. In the present study, the membrane-bound and secreted IgT (mIgT and sIgT) heavy chain genes were cloned for the first time and characterized in flounder (*Paralichthys olivaceus*), and found the nucleic acid sequence were exactly same in the Cτ1–Cτ4 constant domains of mIgT and sIgT, but different in variable regions and the C-terminus. The amino acid sequence of mIgT shared higher similarity with *Bovichtus diacanthus* (51.2%) and *Dicentrarchus labrax* (45.0%). Amino acid of flounder IgT, IgM, and IgD heavy chain was compared and the highest similarity was found between IgT Cτ1 and IgM Cμ1 (38%). In healthy flounder, the transcript levels of IgT mRNA were the highest in gill, spleen, and liver, and higher in peripheral blood leucocytes, skin, and hindgut. After infection and vaccination with *Edwardsiella tarda* via intraperitoneal injection and immersion, the qRT-PCR analysis demonstrated that the IgT mRNA level was significantly upregulated in all tested tissues, with similar dynamic tendency that increased firstly and then decreased, and higher in gill, skin, hindgut, liver, and stomach in immersion than in the injection group, but no significant difference existed in spleen and head kidney between immersion and injection groups. These results revealed that IgT responses could be simultaneously induced in both mucosal and systemic tissues after infection/vaccination via injection and immersion route, but IgT might play a more important role in mucosal immunity than in systemic immunity.

## 1. Introduction

Immunoglobulins (Igs) are humoral mediators produced by B lymphocytes playing a key role in the adaptive immune system of jawed vertebrates to protect the organisms from a wide variety of pathogens. There are two forms of Igs (secreted and membrane-bound form), the soluble Igs exist in body fluids involving in humoral immunity and membrane Igs are antigen receptors of B lymphocyte. Each molecule of Ig is composed of two heavy (H) chains and two light (L) chains, and each chain has a variable region at the N-terminus and one or more constant regions at the C-terminus. The N-terminal regions of both H and L chains are antigen specificity binding sites, while C-terminal regions determine the functional activities and classes of Igs [1,2,3]. H and L chains are encoded by the IgH locus and IgL locus, respectively, wherein translocon configuration and multiple gene segments are mainly responsible for antibody diversity in the IgH locus. The IgH genes’ repertoire is generated by random rearrangement of variable (VH), diversity (DH), and joining (JH) gene segments. Moreover, different constant region genes (CH) selectively perform different effector functions.

According to the amino acid composition and antigenic differences, five IgH isotypes have been identified in mammals: IgM (μ chain), IgD (δ chain), IgG (γ chain), IgA (α chain), and IgE (ε chain). In teleost fish only three isotypes are known to date: IgM, the main antibody presents in the serum, can also be secreted into the skin mucus via the polymeric immunoglobulin receptor (pIgR) [4,5]; IgD, perhaps plays a role in the surveillance and immune response regulation in the fish immune system, and the immune response exhibits certain organization specificity [6,7]; IgT/IgZ (for Trout/Zebrafish), found only in teleosts and thought to be an immunoglobulin specialized in mucosal immunity [8], as observed in the gut, skin, and gill mucus of trout [9,10]. It has been shown IgT exists in serum as monomers and in gut mucus as multimers, similar to the tetrameric IgM [9]. Fish IgM, D, and T/Z classes refer to the protein products of the isotypes μ, δ, and ζ/τ, respectively, which correspond to their associated constant genes. The structure of IgM is highly conserved across species, but IgD and IgT presents diversity. IgD usually contains seven or more CH domains [11]. The basic structure of the ζ/τ gene is not conserved, with species-specific variations across teleosts that the Cζ/τ genes are inserted between VH and DJ genes in zebrafish (*Danio rerio*), fugu (*Fugu rubripes*), and three-spined stickleback (*Gasterosteus aculeatus*) [12,13,14], whereas it is located within the VH gene region in rainbow trout (*Oncorhynchus mykiss*) and Atlantic salmon (*Salmo salar*) [15].

A diversity of the domain number of IgT has been seen among species; there are four constant domains in *O. mykiss* and *D. rerio* IgT/Z [12,15], three constant domains in *G. aculeatus* IgT [13,16], two domains in *F. rubripes* IgZ which corresponding to the first and fourth constant domain of *D. rerio* IgZ, respectively [17]. Carp (*Cyprinus carpio*) and grass carp (*Ctenopharyngodon idellus*) own a similar chimeric variant molecule IgM–IgZ, which consists of the Cμ1 domain and a second domain with the highest similarity to Cζ4 of *D. rerio* [14]. The genome sequence of *C. idellus* IgM–IgZ contains two constant zone exons and two membrane exons, which indicates IgM–IgZ is not composed of IgM and IgZ at the transcription level, but engomphosised at the time of the gene rearrangement. Another new IgZ variant, named IgZ2, has been identified in *C. idellus* and *C. carpio*, which is similar to *D. rerio* IgZ2 [18]. It is interesting to note that these two IgZ variants have different functions, IgZ1 is mainly expressed in systemic tissues against blood pathogens, and the IgZ2 chimera is preferentially expressed in the mucosal compartment to respond to parasite infections [19,20].

In recent years, it has been found that a wide divergence of the IgH chain loci organization exists among teleosts with more and more genomics data excavated [21]. *D. rerio* IgH locus owns its DH and JH gene segments and the Cτ exon is located upstream of Cμ and Cδ exons, in analogy to the mouse T cell receptor α/δ locus [12]. The canonical structure of *O. mykiss* IgH locus is very similar to that of *D. rerio* [15]. The Atlantic salmon (*S. salar*) possesses two parallel IgH isoloci (IGH-A and IGH-B) that can be attributed to its tetraploid past [22]. In addition, Cypriniformes have different types of IgH loci and the two *C. carpio* IgZ sub-isotypes are encoded by two distinct loci [23]. In fugu IgH loci are similarly found upstream of Cμ and Cδ genes and this Ig isotype has its own D and JH segments, but the gene organization differs significantly from *D. rerio* IgZ [17]. In *G. aculeatus* IgH locus Cτ, Cμ, and Cδ exons have been found tandem-duplicated three times and separated by VH, DH, and JH segments. Additionally, a fourth Cτ gene exists at the 3′ end of the locus [13,18]. It is reported that there exist four Ig constant domains constituted in *Thunnus orientalis* IgM and IgT, and three VH gene families are both shared by IgM and IgT among the four VH gene segment families, while another one is solely used by IgM. Moreover, both IgM and IgT use the same DH segment, whereas the JH gene segments are isotype specific [24].

Although IgT has been cloned and characterized at the gene level in a number of bony fish species in the last several years, the biological functions of IgT are not well elucidated [25]. Previous reports demonstrate rainbow trout IgT may play an important role in gut and gill mucosal immunity [9,26], and a similar point is supported by *Megalobrama amblycephala* and *Trematomus bernacchii* [27,28]. Recently it has been found the mucosal adaptive immunity of teleost fish has important roles against aquatic infectious agents; therefore, the study of mucosal immunoglobulins has become an overarching concern. In flounder (*Paralichthys olivaceus*), the existence of tetrameric IgM in the skin mucus has been well documented [4], whereas the IgT gene of *P. olivaceus* has not been conducted, and no information on the function of flounder IgT is available.

*P. olivaceus* is an economically important species in aquaculture industry in China, however, serious diseases, such as bacterial hemorrhagic septicemia caused by *Edwardsiella tarda* infection, have greatly hindered flounder aquaculture [29]. To date, only limited information on the biological function of the innate immune genes of *P. olivaceus* is available [30]. In the present study, the IgT gene of *P. olivaceus* was cloned and characterized, and the distribution of IgT mRNA was detected in a series of tissues (peripheral blood leucocytes, gill, skin, spleen, head kidney, trunk kidney, liver, hindgut, muscle, and stomach) of healthy flounder by real-time reverse transcription PCR (RT-PCR). In order to investigate the biological function of IgT of *P. olivaceus*, the fish was challenged and immunized with live and inactivated *E. tarda* by injection and immersion routes, and the mRNA levels of IgT were determined by quantitative reverse transcription-PCR (qRT-PCR). These data should shed further light on our understanding of the function of IgT in fish adaptive immunity, especially in mucosal immunity, and provide new insights into the vaccine design and vaccination methods in fish aquaculture.

## 2. Results

### 2.1. Molecular Characterization of Flounder IgT

**Figure 1:**
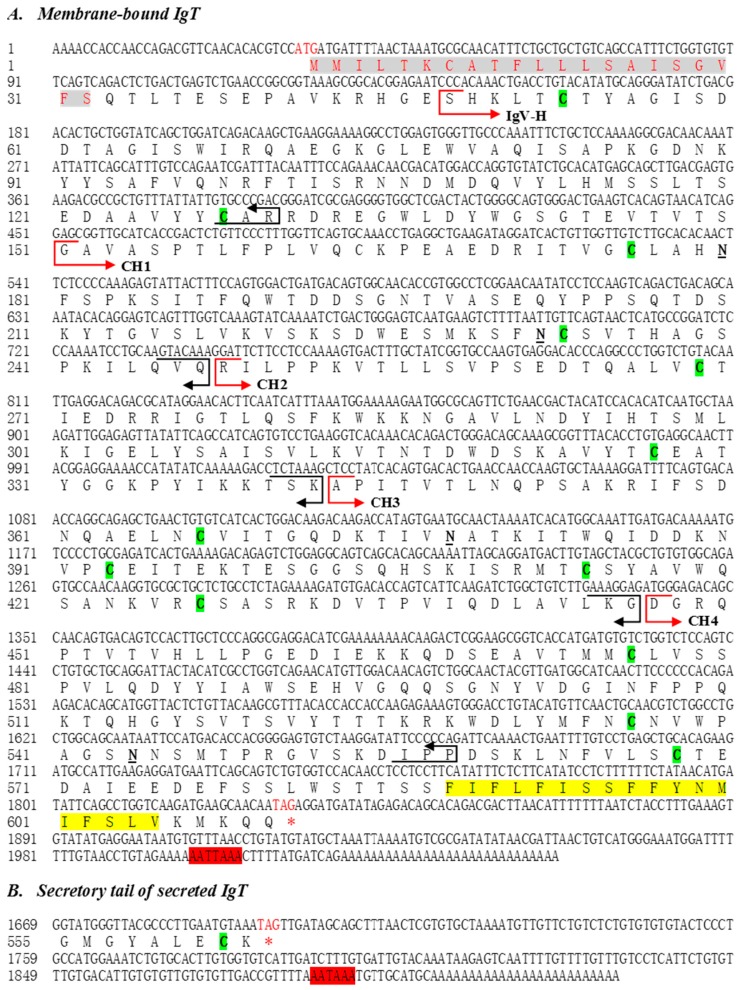
(**A**) Nucleotide and deduced amino acid sequences of the flounder membrane-bound IgT (mIgT, GenBank accession number KX174301). The sequence was divided into VH and four CH domains on the basis of sequence comparisons with the IgH chains of other teleosts. The cysteines (C) were denoted by green bold. The atypical polyadenylation signal (AATTAAA) in the 3′-UTR was shaded in red grey. The signal peptide sequence used hotlink and grey shadow. The four Ig like domains were shown in red arrows for the upstream and dark arrows for the downstream of each domain. The transmembrane region was shaded in yellow grey. Four putative *N*-glycosylation residues were marked with black bold and underline; (**B**) Nucleotide and deduced amino acid sequences of secreted tail part of the flounder secreted IgT (sIgT, GenBank accession number KX174302). The atypical polyadenylation signal (AATAAA) in the 3′-UTR was shaded in red grey.

The full-length cDNA of membrane and secreted form of IgT from flounder were obtained by the rapid amplifications of cDNA ends (RACE) method and submitted to NCBI. The complete sequence of membrane IgT cDNA (mIgT GenBank accession number KX174301) was of 2047 bp, containing an open reading frame (ORF) of 1797 bp, a 5′ UTR of 32 bp, and a 3′ UTR of 215 bp with a putative atypical polyadenylation signal sequence AATTAAA located upstream of the poly (A) tail. The deduced mIgT amino acid sequence encoded a single-spanning transmembrane protein of 599 amino acid residues, which possessed a 21-amino acid signal peptide, an extracellular region, a transmembrane region (TM), and a 51-amino acid intracellular region. The molecular weight of flounder mIgT was 66.61 kDa and pI = 6.99, and four *N*-glycosylation residues on the extracellular region were predicted (Figure 1A). While the secreted IgT cDNA (sIgT GenBank accession nr. KX174302) was composed of 1916 bp, containing an ORF of 1656 bp encoding 552 amino acid residues, and the molecular weight was 60.94 kDa and pI = 7.75 (Figure 1B).

### 2.2. Multiple Sequence Alignment and Phylogenetic Analysis

**Figure 2:**
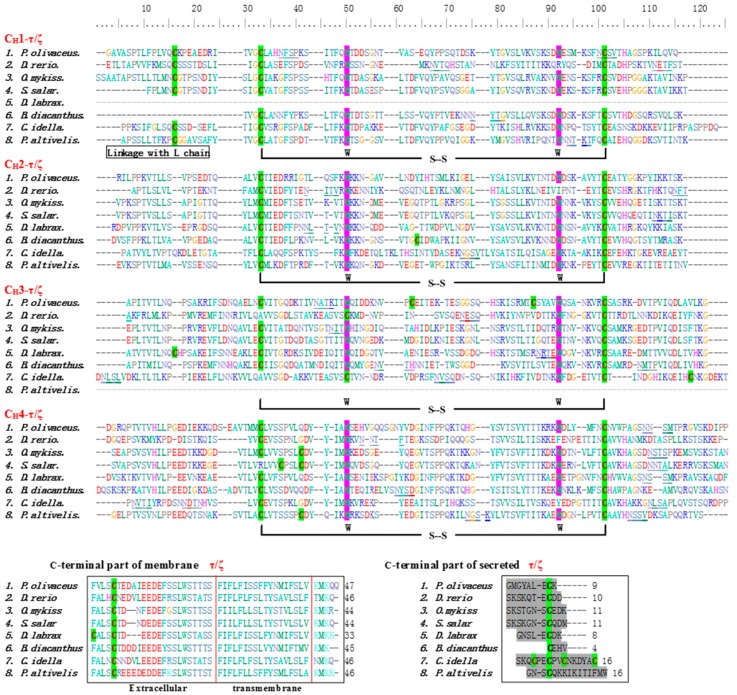
Alignment of translated IgT/IgZ sequences in different fish species. The first alignment showed immunoglobulin constant domain (CH4-τ/ζ–CH4-τ/ζ) and a segment encoding the membrane proximal extracellular part, the transmembrane peptide, and a short cysteine tail. Cysteine and tryptophan residues were shaded green bold and purple bold, and underlined font putative predicted *N*-glycosylation sites were in black bold, respectively. In the second part of the alignment, the C-terminal part of the secreted IgT, which was encoded by the CH4 exon, the letters was shown in the gray shading. GenBank accession numbers for membrane and secreted forms of IgT/Z sequences were displayed: *Paralichthys olivaceus*: KX174301, KX174302; *Danio rerio*: AAT67444.1, AAT67446.1; *Oncorhynchus mykiss*: AAW66980.1, AAW66981.1; *Salmo salar*: ACX50290.1, ACX50293.1; *Dicentrarchus labrax*: AKK32392.1, AKK32388.1; *Bovichtus diacanthus*: AKA09866.1, AKA09828.1; *Ctenopharyngodon idella*: ABY76180.1, ADD82655.1; *Plecoglossus altivelis*: BAP75402.1, BAP75403.1.

**Figure 3:**
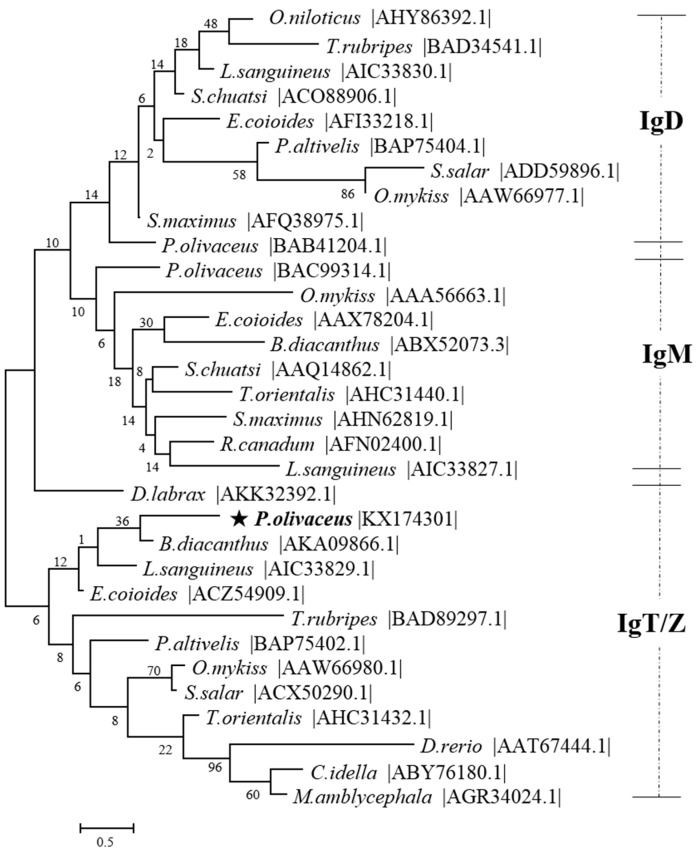
Phylogenetic tree analysis of IgT family members from flounder and other fish species. The five-pointed star highlighted the position of *P. olivaceus*. The tree was constructed by the “neighbor-joining” method using MEGA 5.0 software. Node values represented the percent of bootstrap confidence derived from 1000 replicates. The accession number for each sequence followed the common species name.

The online BLAST analysis showed that the deduced amino acid sequence of IgT (mIgT and sIgT) heavy chain of *P. olivaceus* was constituted by one VH and four Ig-like constant domains (CH1, CH2, CH3, CH4) (Figure 1). Clustal W multiple alignments of flounder IgT revealed that the four constant domains (Cτ1–Cτ4) corresponded to other reported teleost IgT/Z, and nucleic acid sequences of mIgT and sIgT were exactly the same in the Cτ1–Cτ4 constant domains, but different in VH and C-terminus that mIgT contained a C-terminal TM and sIgT owned a short secreted peptide. In addition to the two conserved cysteine and tryptophan residues in VH region and all the CH domains, an extra conserved cysteine residue consisted in CH1 and two additional cysteine residues presented in CH3, as well as an additional conserved cysteine residue existed in the C-terminal region of mIgT and sIgT. Four putative N-linked glycosylation sites, i.e. two in CH1, one in CH3 and one in CH4, were found in IgT of *P. olivaceus* (Figure 2). Protein sequence analysis showed that flounder mIgT presented 51.2% similarity with *Bovichtus diacanthus*, 45.0% with *D. labrax*, 41.4% with *S. salar*, 40.0% with *T. orientalis*, 38.7% with *Epinephelus coioides*, 36.6% with *L. sanguineus*, 35.6% with *O. mykiss*, 25.4% with *D. rerio*, 25.3% with *Plecoglossus altivelis*, 22.5% with *Ctenopharyngodon idella*, 18.1% with *M. amblycephala* and 17.2% with *T. rubripes* (Figure 3). In the constructed phylogenetic tree, the homologous IgM of the teleosts grouped together, IgD clustered together, while IgT/Z clustered together, and IgT of *B. diacanthus*, *L. sanguineus* and *E. coioides* grouped together with *P. olivaceus* (Figure 3).

**Figure 4:**
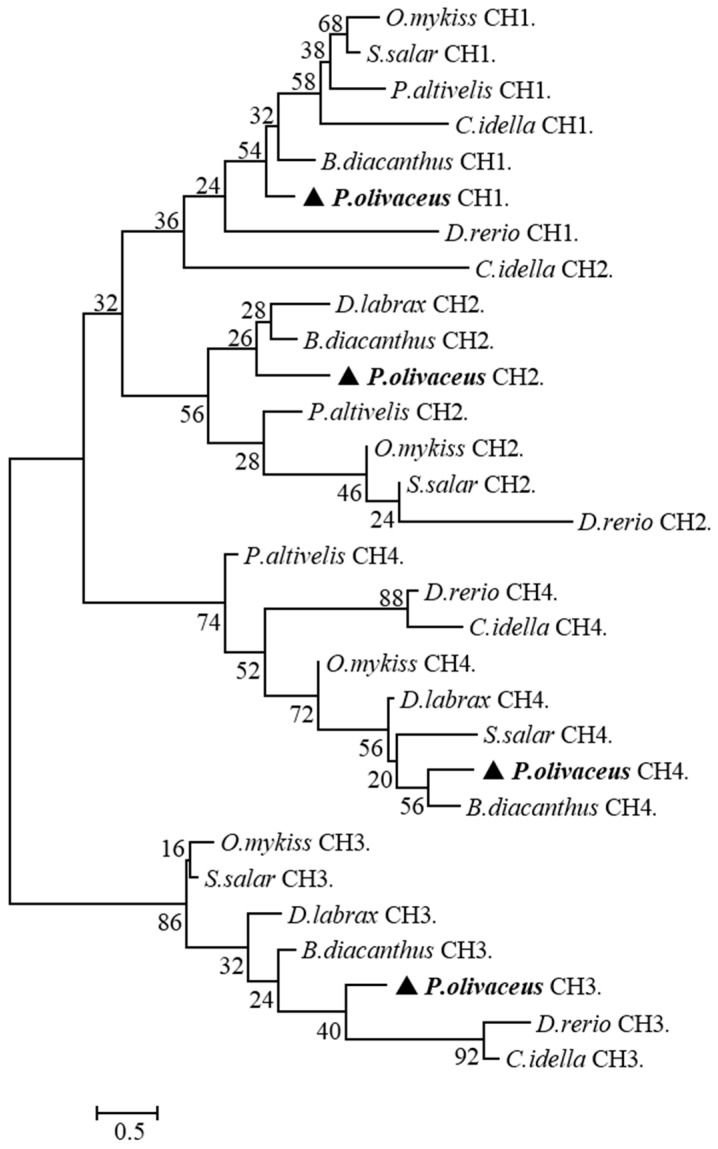
Phylogenetic tree of the single heavy chain constant domains of IgT/Z from eight teleost species reported in Figure 2. The triangle highlighted the position of *P.* *olivaceus*.

As shown in Table 1, the amino acid sequence identity matrix of constant regions and the single constant domains of IgT/Z heavy chain among the different fish species mentioned in Figure 2 were displayed. The constant regions of *P. olivaceus* IgT had the highest similarity to *B.* *diacanthus* (52%), followed by *O.* *mykiss* (43%), *S.* *salar* (42.1%) and *D.* *labrax* (42.2%). Higher similarity in IgT Cτ1 domain was observed between *P. olivaceus* and *B.* *diacanthus* (48.9%), *O.* *mykiss* (43.1%) and *S.* *salar* (43%). For IgT Cτ2 domain, *P. olivaceus* showed higher similarity to *B.* *diacanthus* (52%) and *D.* *labrax* (51%), and then *O.* *mykiss* (40.6%) and *S.* *salar* (36.4%). As compared to the IgT Cτ3 domain of *P. olivaceus* with other fish, the similarity was higher in *D.* *labrax* (55.1%) and *B.* *diacanthus* (52.8%), next in *O.* *mykiss* (42.4%), and *S.* *salar* (43.3%). Moreover, the IgT Cτ4 domain of *P. olivaceus* also shared high similarity to that of *D.* *labrax* (57%) and *B.* *diacanthus* (55.6%), followed by *O.* *mykiss* (45.6%) and *S.* *salar* (43.9%). In the phylogenetic tree constructed, the homologous C_H_1-τ/ζ, C_H_2-τ/ζ, C_H_3-τ/ζ, and C_H_4-τ/ζ clustered together, respectively (Figure 4). In *P. olivaceus*, IgT Cτ1 maintained a higher similarity to Cτ2 (19.1%) and the Cτ2 domain showed higher similarity to Cτ3 (16%). Compared IgT Cτ with IgM Cμ and IgD Cδ domains in *P. olivaceus*, the deduced amino acid sequence of IgT Cτ1 showed highest similarity with that of IgM Cμ1 (38%), followed by IgT Cτ2 (19.1%), and IgD Cδ2 (18.2%) and Cδ7 (17.6%); IgT Cτ2 domain displayed higher similarity with IgM Cμ1 (24.7%) and Cμ2 (19.2%); While IgT Cτ3 domain showed higher similarity with IgD Cδ6 (16%) than other domains and IgT Cτ4 domains showed highest similarity with IgD Cδ7 (16.8%) (Table 2).

### 2.3. Tissue Distribution of IgT Transcript in Healthy Flounder

**Figure 5:**
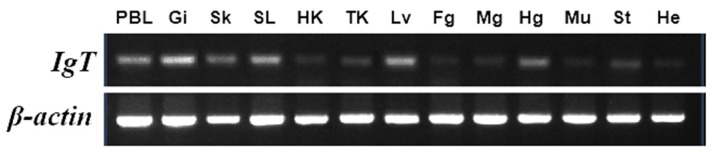
Tissue distribution pattern of IgT mRNA transcripts in healthy flounder. The gene expression profiles of IgT in thirteen tissues were determined by RT-PCR analysis. Individual tissues from three fish were equally pooled for RNA purification. Total RNA from three flounder (*n* = 3) were isolated and subjected to DNase I treatment and then transcribed into cDNA. β-Actin of flounder was employed as an internal reference gene. PBL: peripheral blood leucocytes; Gi: gill; Sk: skin; SL: spleen; HK: head kidney; TK: trunk kidney; Li: liver; Fg: foregut; Mg: midgut; Hg: hindgut; Mu: muscle; St: stomach; He: heart.

The expression of IgT mRNA was found in all tested tissues of flounder (Figure 5). The results showed that IgT expression was detected at the highest level in gill, spleen, and liver, and higher levels in peripheral blood leukocyte, skin, and hindgut, followed by stomach, head kidney, trunk kidney, midgut, and foregut, but much lower in muscle and heart.

### 2.4. Tissue Expression of IgT Post Bacterial Challenge

**Figure 6:**
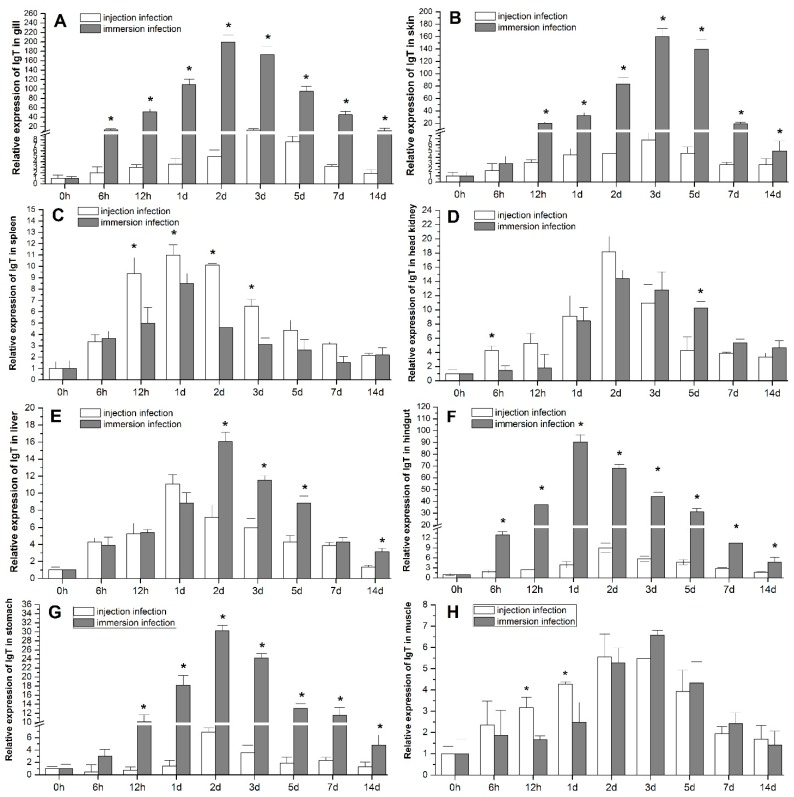
The gene expression profiles of IgT in different tissues ((**A**): gill, (**B**): skin, (**C**): spleen, (**D**): head kidney, (**E**): liver, (**F**): hindgut, (**G**): stomach, (**H**): muscle) of flounder were determined by qRT-PCR analysis post-infection by intraperitoneal (IP) injection with live *E.* *tarda* bacteria (0.2 mL; 1.0 × 10^7^ CFU/mL per fish) and immersion (1.0 × 10^8^ CFU/mL bath for 60 min), respectively. Individual tissue from three fish was equally pooled for RNA purification. 18sRNA of flounder was employed as an internal reference gene. Values were presented as means ± standard deviation (*n* = 3), and the asterisk (*) indicated the significant differences (*p* < 0.05) between IP injection group and immersion group at the each time points after infection.

The results of qRT-PCR examination showed that the expression of IgT transcript increased significantly in the eight tissues tested in the bacteria-challenged groups compared with the blank control and the expression level before challenge (0 h) (Figure 6), while the expression of IgT in the negative control had no significant difference from that of the blank control during experiment (the results not shown). The expression of IgT mRNA was significantly up-regulated post *E. tarda* infection by immersion and IP injection, and showed a tendency to increase firstly and then to recover to blank control level after 14 days, with a peak at 1–3 days post infection (p.i.). The up-regulation of IgT expression was significantly higher in the immersion group than the injection group from 6 h p.i. in gill (Figure 6A), skin (Figure 6B), hindgut (Figure 6F) and stomach (Figure 6G), and from 2 days p.i. in liver (Figure 6A). While in spleen (Figure 6C), the up-regulation of IgT in immersion group was significantly lower than injection group from 12 h p.i. Compared with the control, the peak values of IgT expression was 199-fold in gill, 159-fold in skin, 90-fold in hindgut, 30-fold in stomach, 16-fold in liver, 14-fold in head kidney, eight-fold in spleen, and 6.5-fold in muscle in immersion group; and 18-fold in head kidney, 12-fold in gill, 11-fold in spleen and liver, nine-fold in hindgut, 6.8-fold in stomach, six-fold in skin, and 5.5-fold in muscle in injection group, respectively (Figure 6A–H). Changes in peak expression of IgT in immersion group, in contrast to injection group, were most dramatic in skin (26-fold, three days p.i.), gill (16.7-fold, 2–3 days p.i.) and hindgut (10-fold, one day p.i.), followed by stomach (4.2-fold, two days p.i.), and then liver (1.45-fold, 1–2 days p.i.) and muscle (1.2-fold, 2–3 days p.i.), however, lower in spleen (0.72-fold, one day p.i.) and head kidney (0.78-fold, two days p.i.).

### 2.5. Expression of IgT after Inactivated E. tarda Immunization

**Figure 7:**
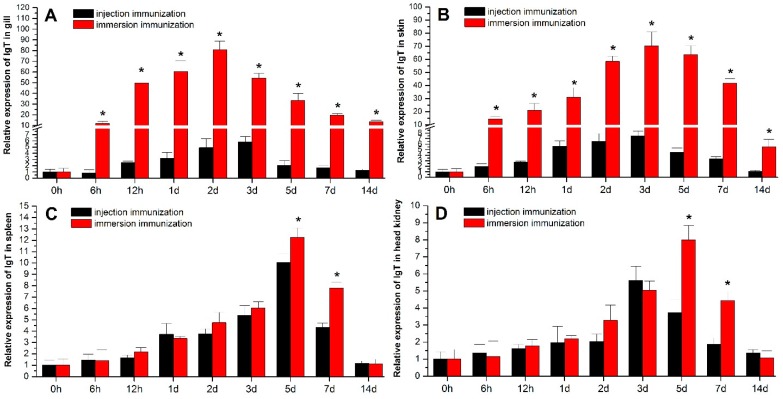

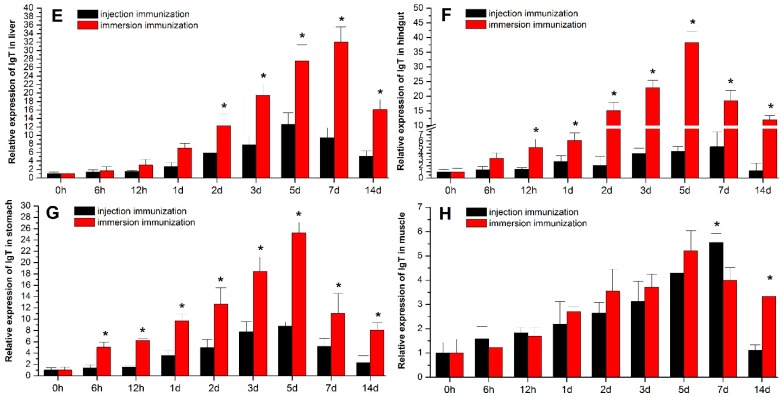
The dynamic changes of IgT mRNA transcripts expression in different tissues ((**A**): gill, (**B**): skin, (**C**): spleen, (**D**): head kidney, (**E**): liver, (**F**): hindgut, (**G**): stomach, (**H**): muscle) of flounder were determined by qRT-PCR assay post-immunization by IP injection with formalin-killed *E.* *tarda* bacteria (0.2 mL; 1.0 × 10^8^ CFU/mL per fish) and bath immersion (1.0 × 10^8^ CFU/mL for 60 min), respectively. Individual tissues from three fish were equally pooled for RNA purification. 18sRNA of flounder was employed as an internal reference gene. The identities of all PCR products were confirmed by sequencing. Values were presented as means ± standard deviation (*n* = 3), and the asterisk (*) indicated the significant differences (*p* < 0.05) between IP injection group and immersion group at each time points after immunization.

The qRT-PCR results showed that IgT mRNA expressions were up-regulated significantly compared to the blank control in all tested tissues after IP injection and immersion immunization with formalin-killed *E. tarda* (Figure 7), displaying a similar tendency that first increased and then decreased. However, the expression levels were down to the blank control level (at 14 days p.i.) earlier in injection than in immersion group. The up-regulations of IgT expression were significantly higher in gill (Figure 7A), skin (Figure 7B), liver (Figure 7E), hindgut (Figure 7F), and stomach (Figure 7G) in immersion group than injection group from 6 h to 14 days, and reached peak earlier in gill and skin (2–3 days) than in liver, hindgut, and stomach (5–7 days), whereas in spleen, head kidney, and muscle (Figure 7C,D,H), the two immunization routes induced similar dynamic changes of IgT expression, and the peak time appeared at 5–7 days. Compared with the control, The peak value of IgT expression was 80.2-fold in gill, 58.5-fold in skin, 38.2-fold in hindgut, 32.3-fold in liver, 25.7-fold in stomach, 12.4-fold in spleen, 5.6-fold in head kidney, and 5.2-fold in muscle in the immersion group; and 12.6-fold in liver, 10.5-fold in spleen, 8.7-fold in stomach, 7.5-fold in skin, 5.8-fold in gill, 5.7-fold in head kidney, 5.5-fold in muscle, and 5.1-fold in hindgut in the injection group, respectively (Figure 7A–H). The peak expression of IgT in immersion group, in contrast to injection group, were most dramatic in gill (13.8-fold), skin (7.7-fold), and hindgut (7.6-fold), followed by stomach (2.8-fold) and liver (2.5-fold), but no significant difference existed in spleen (1.2-fold), head kidney (0.89-fold), and muscle (0.94-fold) between the two immunization groups.

## 3. Discussion

The immunoglobulin of teleost fish has important immunological roles against aquatic infectious agents. Recent studies indicate that IgM is the principal immunoglobulin involved in humoral immunity and the primordial mucosal Ig, IgD potentially performs a unique role in vertebrate immune responses, and IgT acts as a specialized mucosal Ig in bony fish [6,31,32,33,34,35]. The existence and biological functions of IgM and IgD has been well documented in *P. olivaceus* [36,37], but IgT has not been reported. The present study reported for the first time the membrane-bound and secreted forms of IgT genes encoding the heavy chain constant region in *P. olivaceus*, characterized the nucleotide and protein sequence, and analyzed its tissue distribution in healthy fish, also firstly demonstrated the immune response pattern in the process of infection and immunization by *E. tarda*, and determined its functions in the systemic and mucosal immunity of flounder.

Sequence analysis revealed that the primary and secondary structure of the flounder mIgT shared a high degree of sequence similarity and phylogenetic relationship to the mIgT/Z of other teleost fish, presenting 51.2% similarity with *B. diacanthus*, 45.0% with *D. labrax*, 41.4% with *S. salar*, 40.0% with *T. orientalis*, 38.7% with *E. coioides*, 36.6% with *L. sanguineus*, 35.6% with *O. mykiss*, and 25.4% with *D. rerio*. It is reported that there exists great variation in the structure of the IgT heavy chain gene among fish species, and the IgT heavy chain of most fish consists of VH, DJ area, and two to four Ig tau constant domains [8,13,16]. The present study found that the prototypic gene structure of the flounder IgT (mIgT and sIgT) consisted of one VH, four Ig-like constant domains (CH1, CH2, CH3, and CH4), and a C-terminus, which corresponded to IgT of trout [8], and the nucleic acid sequences of mIgT and sIgT were exactly the same in the Cτ1–Cτ4 constant domains, but different in the VH and C-terminus, the mIgT heavy chain constant region was followed by a TM and sIgT followed by a short secreted peptide. The difference in gene structure between mIgT and sIgT might indicate their different functions in the immune response, and more research on their biological features is needed. Moreover, each Ig-like constant area of flounder IgT contained two cysteine and tryptophan conservative sites, which might be involved in the formation of disulfide bonds and play a decisive role in the formation and maintenance of the immunoglobulin spatial structure [38]. Additionally, an extra conserved cysteine residue in CH1 might play an important role in connecting the H chain to L chain, and two additional cysteine residues in CH3 might assist in connecting the H chain to another H chain or forming a polymer structure. Generally, some transcription regulatory elements localized in 5′-UTR could influence the translation of gene expression, the sequence of 5′-UTR were short in the present sequences, similar to sIgZ of *M. amblycephala* (KC894946), which might indicate some motif in the 5′-UTR that provided a link between transcription and post-transcriptional stages of IgT/Z gene expression; another possibility was that the 5′-UTR sequence nonspecifically combined with the adapter primer during the first-strand cDNA reverse transcription of RACE [27,39]. 3′-UTR usually plays a vital role of mediation and stabilization in most genes’ translation, and the sequences are highly diverse [40,41]. It is worth noting that the 3′-UTR sequences of flounder mIgT/sIgT were short with an integrity poly (A) tail, similar to *L. sanguineus* (KF728201.1), *E. coioides* IgZ (GU182366.1), and *T. bernacchii* IgT (KP876587), and the full-length have been verified correct, demonstrating there would be no unspecific priming during the PCR. Furthermore, it was reported that the average length of 3′-UTR sequences increased during evolution and their utilization associated to organism complexity [42], thus, we speculated that there existed species specificity in the 3′-UTR sequence of IgT/Z.

The constant regions (CH1–CH4) of *P. olivaceus* IgT showed the highest similarity with *B.* *diacanthus* (52%), followed by *O.* *mykiss* (43%), *S.* *salar* (42.1%), and *D.* *labrax* (42.2%). Interestingly, a similar identity was observed as compared single *P. olivaceus* IgT Cτ1, Cτ2, Cτ3, and Cτ4 domains with other fish species, which suggested an equivalent evolution extent among each Cτ domain of *P. olivaceus*. The domains of IgM (AB052744, μ1–μ4) and IgD (AB052658, δ1–δ7) heavy chain of *P. olivaceus* were analyzed by Srisapoome et al [37], but the amino acid similarity among each domain of IgM, IgD, and IgT was not compared. In the present study, domain by domain comparison of IgT (τ1–τ4), IgM (μ1–μ4) and IgD (δ1–δ7) heavy chain was performed and displayed that the amino acid identity was highest between IgT Cτ1 and IgM Cμ1 (38%), followed by IgT Cτ2 and IgM Cμ1 (24.7%), then decreased gradually between other single constant domains, and the data indicated that Cτ3 and Cτ4 provided the specificity to the IgT isotype in flounder. Relatively lower similarity between IgD Cδ domains and IgM Cμ, IgT Cτ was observed, which might suggest that the evolution level of IgD was different from IgM and IgT, and forebode difference of biological function among them [38].

The expression and distribution patterns of IgT have certain tissue specificity, but still exhibit considerable variation in different fish species. The zebrafish IgZ was mainly expressed in lymphoid organs, including thymus [12]. IgT mRNA levels are higher in the spleen, head kidney, trunk kidney, and gill in *P. altivelis* [43]. A gene similar IgT in fugu and mandarin (*Siniperca chuatsi*) fish was expressed in head kidney, spleen, and gill [17,33]. In trout and salmon, IgT displayed similar expression in various tissues, especially in spleen and head kidney [15,44]. The present study showed that the IgT expression was highest in gill, spleen and liver, and higher in leukocyte, skin, and hindgut, followed by stomach, head kidney, trunk kidney, midgut, and foregut, and lower in the stomach and muscle in healthy flounder. The spleen and head kidney of bony fishes are predominant systemic immune organs, which are functionally and morphologically similar to the bone marrow of mammals [45]. It is verified that the gill, skin, hindgut and liver are important mucosa-associated lymphoid tissues of teleosts which are abundant with immune cells such as B and T cells, macrophages and granulocytes [46]. Therefore, the higher expression of IgT in these systemic and mucosal immune tissues in flounder suggested that IgT might play a vital role in the systemic and mucosal immunity.

The administration of intraperitoneal injection infection or vaccination mostly induced a fish systemic immune response, whereas the immersion rout is always thought to activate the mucosal immune response by the mucosal tissues, such as gill, skin, and hindgut [47,48,49]. In order to determine the role of flounder IgT in the systemic and mucosal immune regulation, the fish were infected and vaccinated with live and inactivated *E. tarda* by intraperitoneal injection and immersion, respectively, and the results showed that the IgT mRNA level was up-regulated significantly in all tested tissues, with similar dynamic tendency that increased firstly and then decreased, which revealed that IgT could participate in the immune response of flounder against bacterial pathogens, and both injection and immersion infection/immunization routes all could simultaneously induce systemic and mucosal immune responses. A good example was shown in rainbow trout that the IgT was not only involved in mucosal immunity, but also in systemic immunity [9,50]. Furthermore, the up-regulation of IgT in gill, skin, liver, hindgut, and stomach were obviously higher in immersion than injection groups after infection and vaccination, indicating that IgT might play more important role in the mucosal immune than systemic immune in flounder. On the other hand, no significant difference in IgT up-regulation expression was found in spleen and head kidney between immersion and injection group, suggesting that both immersion and injection routes could induce similar IgT response in systemic immune organs of flounder.

In flounder, IgM has been verified to play important roles in the mucosal immunity. We have previously developed the monoclonal antibodies (McAb) of anti-serum IgM and anti-mucus IgM of flounder, and found that the IgM antibody levels in serum and mucus increased post-immunization in flounder [51,52,53,54]. Moreover, a secreted component-like molecule (SC) of pIgR that mediated Igs secretions was detected in skin mucus of flounder using the polyclonal antibodies against pIgR, suggesting flounder IgM involved in mucosal immunity [5]. Therefore, further research to illuminate the biological function and transport mechanism of IgT and the functional relationship of IgT and IgM in mucosal immunity in *P. olivaceus* are needed.

In conclusion, the membrane-bound and secreted forms of IgT in flounder were cloned and its expression analyses were performed in this study. The IgT mRNA level was up-regulated after *E. tarda* infection and vaccination via the IP injection and immersion routes, and the up-regulation of IgT was significantly higher in gill, skin, liver, hindgut, and stomach in immersion than injection group, but similar in spleen and head kidney in both immersion and injection groups. These results suggested that this unique IgT could participate in the immune response of flounder against bacterial pathogens in systemic and mucosal tissues simultaneously, but might play more important roles in mucosal immunity than systemic immunity. To our knowledge, this was the first study which revealed the immune response pattern of IgT in flounder after infection/vaccination via injection and immersion routes, which are of potential importance for the immunological control of fish disease. More importantly, these results have crucial implications for the future design of fish vaccines that stimulate systemic, as well as mucosal, immunity. However, more research on the flounder IgT in the protein expression level are needed in the future.

## 4. Materials and Methods

### 4.1. Ethics Statement

This study was carried out strictly in line with the procedures in the Guide for the Use of Experimental Animals of the Ocean University of China. In this study, for the methods used in animal experiments were approved by the Institutional Animal Care and Use Committee of Ocean University of China (Permit Number: 20150101). All efforts were dedicated to minimize suffering.

### 4.2. Fish

Healthy flounder (average length: 15–17 cm) were obtained from a fish farm in Shandong Province, Rizhao, China. The fish were kept in quarantined plastic tanks with a follow-through water volume of 500 L (dissolved oxygen 6.0 ± 0.5 mg·L^−1^; temperature 20 ± 1 °C) and fed with commercial diet twice daily. All fish were acclimatized for two weeks prior to treatment.

### 4.3. Cloning of the Full-Length IgT

Three apparently healthy flounder were sampled, and the gill, spleen, head kidney, and hindgut were isolated immediately after anesthetization with MS-222, frozen in liquid nitrogen and stored at −80 °C prior to use. The tissue was immediately homogenized in TRIzol Reagent (Invitrogen, CarIsbad, CA, USA), and total RNA was extracted using the E.Z.N.A. HP Total RNA Kit (Omega Bio-Tek, Norcross, GA, USA) and reverse-transcripted into first-strand cDNA using SuperScript Reverse Transcriptase according to the manufacturer’s instructions. By performing a homology search of the common flounder genome using the *O.* *mykiss* (AAW66980.1) with BLAST [55], IgT-like sequences were obtained. For the isolation of the flounder IgT cDNA fragment, multiple alignments of the amino acid sequences of several other fish were performed using the DNAMAN multiple sequence alignments program. Degenerate primers were designed based on the highly conserved sequences of *T.* *orientalis* (KF713336.1), *Lutjanus sanguineus* (KF728201.1), and *Dicentrarchus labrax* (KP096356.1). A flounder IgT partial gene sequence was cloned using degenerate primers IgT-F/IgT-R from the common flounder cDNA template. PCR amplification was performed under the following conditions: one cycle of 94 °C for 5 min, 35 cycles of 94 °C for 30 s, 56 °C for 30 s, and 72 °C for 50 s, and a final extension of 72 °C for 10 min. The PCR products were gel-extracted and ligated into the pMD-18T vector. Following transformation into competent *Escherichia coli* DH5α cells, positive clones were screened by Ampicilin selection and colony PCR and sequenced by Shanghai BioSune Biotechnology Co., Ltd. (Shanghai, China).

Subsequently, rapid amplifications of cDNA ends (RACE) were performed using the SMART RACE cDNA amplification Kit (BD Bioscience Clontech Co., Palo Alto, CA, USA) to get the full-length IgT cDNA sequences by the specific primers which were designed based on the obtained partial sequence. For 3′ RACE, the first-strand cDNA was performed as described above with the adapter primer 3′-CDS supplied by the kit. The first round of PCR was performed using the primer pair of IgT 3′-RACE 1st out /UPM (adaptor primer), under the following conditions: one cycle of 94 °C for 5 min, five cycles of 94 °C for 30 s, 64 °C for 30 s, and 72 °C for 2 min, 29 cycles of 94 °C for 30 s, 60 °C for 30 s, and 72 °C for 2 min, followed by a final extension of 72 °C for 10 min. The resultant products were diluted (1:20) and re-amplified in the second round PCR using the primer pair of IgT 3′-RACE nested in /NUP, under the same reaction conditions. For 5′-RACE, the first-strand cDNA was synthesized using oligo d(T)-anchor primer and 5-AP-DG-adapter primer. A primary PCR was accomplished with antisense primer IgT 5′-RACE 1st and sense primer UPM. A nest gene-specific primer IgT 5′-RACE nested and the manufacturer’s PCR anchor primer NUP were used to accomplish PCR amplification of the double-stranded cDNA. Amplification cycles were performed as follows: one cycle of 94 °C for 5 min, 35 cycles of 94 °C for 30 s, 58 °C for 30 s, and 72 °C for 2 min, followed by a final extension of 72 °C for 10 min. All primers used for this cloning process were listed in Table 3.

### 4.4. Domain Organization and Sequence Analyses

The full length of membrane-bound IgT (mIgT) and secreted IgT (sIgT) were confirmed by RT-PCR using sequence-specific primers qIgT-F, mIgT-R, and sIgT-R (Table 3). The open reading frames (ORFs) and deduced protein sequences of mIgT and sIgT were predicted using an ORF Finder program [56], and also by blasting genomic stretches against protein databases at NCBI (blastx). Immunoglobulin domains were predicted by PROSITE database [57]. The trans-membrane domain was predicted using the TMpred program [58], and N-linked glycosylation sites were predicted by the NETNGLYC 1.0 server [59]. The draft genome databases and expressed sequence tag (EST) databases distributed at Swiss-Prot protein databases, Expasy, Ensembl, UCSC Genome Browser and TIGR were employed to retrieve the immunoglobulin molecules. Multiple alignments of sequences were conducted using the Clustal W program (version 1.83) [60]. On the basis of the alignment, phylogenetic trees were constructed with MEGA 5 software using the neighbor-joining method [61]. Statistical significance of each branch was examined by the bootstrap. The veracity of these trees was studied using the bootstrapping method by executing 1000 replicates. The isoelectric point (pI), potential *N*-glycosylation sites and the molecular weight of the deduced IgT proteins were calculated by the Expert Protein Analysis System [62].

### 4.5. Quantitative Analysis of IgT Expression at the mRNA Level in Healthy Flounder

The expression of IgT mRNA in tissues of healthy fish, including peripheral blood leucocytes, gill, skin, spleen, head kidney, trunk kidney, liver, foregut, midgut, hindgut, muscle, stomach, and heart, were detected by RT-PCR. Total RNA was extracted from different tissues as described in 2.2 and the RNA concentration and integrity were measured using NanoDrop ND-1000 (NanoDrop Technologies, Wilmington, DE, USA) and by agarose gel electrophoresis. First-strand cDNA was synthesized using a PrimeScript RT reagent kit (Takara, Otsu, Japan) with Oligo dT/random hexamer primers, according to the manufacturer’s protocol. cDNA was diluted in diethyl dicarbonate (DEPC)-treated water and stored at −20 °C. Two gene-specific primers IgT1-F and IgT1-R (Table 3) were used to amplify a 537 bp gene fragment of IgT, and two actin primers β-ActinF and β-ActinR (Table 3) were used to amplify β-actin gene fragment as the internal control for RT-PCR. PCR amplification was performed under the following conditions: one cycle of 94 °C for 5 min, 35 cycles of 94 °C for 30 s, 56 °C for 30 s, and 72 °C for 60 s, followed by a final extension of 72 °C for 10 min. The PCR products were gel-extracted and pictures were taken. The cloned amplicons were confirmed by sequencing. Primer sequences were given in Table 3.

### 4.6. qRT-PCR Analysis of IgT Expression in Response to Live and Inactivated E. tarda

Healthy flounder were divided into six experimental groups and transferred into tanks (50 fish/tank) that each contained 500 L of water. The bacterin of live and inactivated *E. tarda* was prepared as described previously [54]. Group A was infected by intraperitoneal (IP) injection with live *E. tarda* (0.2 mL; 1.0 × 10^7^ colony-forming units (CFU)/mL in phosphate buffer saline (PBS)); group B was infected by immersion with live *E. tarda* (1.0 × 10^8^ CFU/mL) for 60 min; group C was immunized by IP injection with formalin-killed *E. tarda* (0.2 mL; 1.0 × 10^8^ CFU/mL); group D was vaccinated by immersion with formalin-killed *E. tarda* (1.0 × 10^8^ CFU/mL) for 60 min; group E was injected with equal dose PBS as negative control; and group F was not treated as blank control. The gill, skin, spleen, head kidney, liver, hindgut, stomach, and muscle were collected from three fish from each group before treatment (0 h) and at 6 h, 12 h, 1 day, 2 days, 3 days, 5 days, 7 days and 14 days post-treatment, immediately transferred to a −80 °C freezer, and used to isolate total RNA. Total RNA and cDNA were prepared from tissue samples as previously described in Section 2.4. qRT-PCR was performed using a Roche480 real-time PCR system (LightCycler480, Agilent, Santa Clara, CA, USA). Each qPCR was performed in a total volume of 20 μL containing 10 μL of SYBR Green I Master, 0.4 μL of forward and reverse primers (10 mM), 2 μL of diluted cDNA and RNase-free water. The thermal cycling profile consisted of an initial denaturation at 95 °C for 30 s, followed by 45 cycles of denaturation at 95 °C for 5 s and annealing/extension at 60 °C for 30 s. An additional temperature-ramping step was utilized to produce melting curves of the reaction from 65 to 95 °C. The expression level of IgT in blank control individuals was defined as 1. A 150 bp gene fragment of IgT was amplified by the specific primers IgT2-F and IgT2-R (Table 3), and two primers 18sRNA-F and 18sRNA-R (Table 3) of flounder were used to amplify 18sRNA gene fragment as the internal control for qRT-PCR. The identities of all the products were confirmed by sequencing.

### 4.7. Statistical Analysis

The qRT-PCR data were analyzed using MX Pro-Mx3000P Multi-plex Quantitative PCR system Software (LightCycler480, Agilent) and the relative expression ratio (*R*) of mRNA was calculated according to the formula 2^−^^ΔΔ*C*t^ [63]. The data were normalized for each gene against those obtained for 18sRNA [64]. The results were presented as means with standard deviations of fold increase/decrease from three fish at each time point. The transcript level of each Ig gene was compared between different treatment at each time point by one-way ANOVA, and the level of significance was set to *p* < 0.05 for all analyses. The statistical software Origin (Version 8.0, OriginLab, Northhampton, MA, USA) was used for creating graphs.

## Figures and Tables

**Table 1:** Amino acid sequence identity matrix of constant regions and the single constant domains of IgT/Z heavy chain between the different fish species reported in Figure 2.

	1. *P. olivaceus*	2. *D. rerio*	3. *O. mykiss*	4. *S. salar*	5. *D. labrax*	6. *B. diacanthus*	7. *C. idella*	8. *P. altivelis*
**Constant regions (CH1–CH4)**								
**1.** ***P. olivaceus***		●22.3	●43	●42.1	●42.2	●52	●23.5	●25.4
**2.** ***D. rerio***			●24.5	●23.2	●17.9	●19.3	●36.3	●17.6
**3.** ***O. mykiss***				●79.8	●31.6	●39	●27.5	●34.1
**4.** ***S. salar***					●33.1	●39.5	●26.2	●33
**5.** ***D. labrax***						●44.3	●17.3	●17.7
**6.** ***B. diacanthus***							●21.5	●25.8
**7.** ***C. idella***								●20.5
**8.** ***P. altivelis***								
**C_H_1-τ/ζ**								
**1.** ***P. olivaceus***		●23.2	●43.1	●43.0		●48.9	●28.0	●35.0
**2.** ***D. rerio***			●16.6	●17.0		●17.3	●18.8	●20.2
**3.** ***O. mykiss***				●79.8		●37.2	●34.8	●48.0
**4.** ***S. salar***						●39.1	●36.5	●47.4
**5.** ***D. labrax***								
**6.** ***B. diacanthus***							●27.1	●31.9
**7.** ***C. idella***								●32.6
**8.** ***P. altivelis***								
**C_H_2-τ/ζ**								
**1.** ***P. olivaceus***		●23.7	●40.6	●36.4	●51.0	●52.0	●23.1	●34.3
**2.** ***D. rerio***			●35.4	●34.3	●27.8	●28.8	●16.1	●31.2
**3.** ***O. mykiss***				●88.4	●42.7	●38.5	●19.4	●42.1
**4.** ***S. salar***					●42.7	●36.4	●17.5	●41.0
**5.** ***D. labrax***						●58.9	●19.4	●34.3
**6.** ***B. diacanthus***							●20.3	●38.5
**7.** ***C. idella***								●20.3
**8.** ***P. altivelis***								
**C_H_3-τ/ζ**								
**1.** ***P. olivaceus***		●21.6	●42.4	●43.3	●55.1	●52.8	●18.1	
**2.** ***D. rerio***			●22.6	●20.7	●23.3	●19.8	●47.8	
**3.** ***O. mykiss***				●83.9	●37.9	●40.5	●21.5	
**4.** ***S. salar***					●39.8	●44.3	●22.4	
**5.** ***D. labrax***						●54.2	●18.6	
**6.** ***B. diacanthus***							●15.5	
**7.** ***C. idella***								
**8.** ***P. altivelis***								
**C_H_4-τ/ζ**								
**1.** ***P. olivaceus***		●26.7	●45.6	●43.9	●57.0	●55.6	●24.1	●36.7
**2.** ***D. rerio***			●30.6	●27.9	●25.6	●17.7	●52.3	●23.2
**3.** ***O. mykiss***				●78.7	●45.6	●39.3	●33.6	●46.3
**4.** ***S. salar***					●45.6	●37.6	●31.8	●43.6
**5.** ***D. labrax***						●51.3	●26.3	●33.6
**6.** ***B. diacanthus***							●19.4	●32.2
**7.** ***C. idella***								●30.3
**8.** ***P. altivelis***								

Amino acid sequence identities with decreasing degree were indicated by four icon sets ●●● ●.

**Table 2:** Amino acid sequence identity matrix of the single constant domains of IgT (KX174301, τ1–τ4), IgM (AB052744, μ1–μ4) and IgD (AB052658, δ1–δ7) heavy chain in *Paralichthys olivaceus*.

	τ1	τ2	τ3	τ4	μ1	μ2	μ3	μ4	δ1	δ2	δ3	δ4	δ5	δ6	δ7
**τ1**		●19.1	●12.0	●13.2	●38.0	●14.7	●11.9	●12.6	●13.2	●18.2	●10.1	●15.2	●11.9	●13.7	●17.6
**τ2**			●16.0	●14.0	●24.7	●19.2	●8.10	●13.3	●21.6	●12.3	●8.30	●13.5	●11.8	●15.7	●16.6
**τ3**				●12.9	●11.1	●13.5	●12.7	●10.7	●11.1	●13.0	●12.7	●12.3	●14.2	●16.0	●14.8
**τ4**					●13.2	●9.20	●13.9	●12.8	●8.80	●10.6	●9.50	●14.1	●14.7	●14.2	●16.8
**μ1**						●15.6	●12.8	●13.4	●16.0	●19.2	●16.6	●15.2	●14.6	●15.5	●14.7
**μ2**							●9.20	●10.6	●14.6	●10.2	●11.9	●14.1	●11.7	●11.3	●13.1
**μ3**								●13.8	●10.0	●15.2	●11.2	●12.2	●13.7	●12.5	●11.9
**μ4**									●13.2	●14.0	●12.4	●15.0	●18.4	●20.0	●15.0
**δ1**										●14.1	●12.8	●15.0	●12.8	●18.3	●17.9
**δ2**											●10.2	●11.5	●17.4	●9.00	●11.5
**δ3**												●16.3	●14.6	●13.6	●10.3
**δ4**													●13.7	●19.6	●17.4
**δ5**														●16.0	●14.6
**δ6**															●16.6
**δ7**															

Amino acid sequence identities with decreasing degree were indicated by four icon sets ●●● ●.

**Table 3:** Primers used for gene cloning and qPCR in this study.

Primer Name	Oligonucleotide Sequence
Core segment PCR	
IgT-F	5′-ACAGTGACACTGAAMCMWCCAAGT-3′
IgT-R	5′-GTYACMAGYGTYTWCACCATC-3′
RACE PCR	
IgT 3′-RACE 1st	5′-CCCTACTGACCTTTAAGCCA-3′
IgT 3′-RACE nested	5′-CGATCACCAATGTTCACTGC-3′
IgT 5′-RACE 1st	5′-AGGCAGCACAGGTAACAGGT-3′
IgT 5′-RACE nested	5′-TTGGTGTGTATGCTGAAAGAT-3′
3′-CDS	5′-AAGCAGTGGTATCAACGCAGAGTAC(T)30VN-3′
UPM	5′-CTAATACGACTCACTATAGGGCAAGCAGTGGTATCAACGCAGAGT-3′
NUP	5′-AAGCAGTGGTATCAACGCAGAGT-3′
5-AP-DG	5′-AAGCAGTGGTATCAACGCAGAGTACGCGGGGGGGGGG-3′
Full-length validation	
qIgT-F	5′-CCACCAACCAGACGTTCAAC-3′
mIgT-R	5′-TTGCTTCATCTTGACCAGGC-3′
sIgT-R	5′-GGCGTAACCCATACCCTTAG-3′
RT-PCR	
IgT1-F	5′-CGACTCTGTTCCCTTTGGTTC-3′
IgT1-R	5′-GTTTTCCTCCGTAAGTTGCCT-3′
qRT-PCR	
IgT2-F	5′-TAATTGTTCAGTAACTCATGCCG-3′
IgT2-R	5′-GATTGAAGTGTTCCTATGCGTCT-3′
β-actin F	5′-AGAGCAAGAGAGGCATCCTGAC-3′
β-actin R	5′-CGATGGGTGATGACCTGTCC-3′
18sRNA-F	5′-GGTCTGTGATGCCCTTAGATGTC-3′
18sRNA-R	5′-AGTGGGGTTCAGCGGGTTAC-3′

RACE, rapid amplification of cDNA ends; Ig, immunoglobulin; qRT, quantitative real time; F, forward; R, reverse.
